# Cardiac and renal function in a large cohort of amateur marathon runners

**DOI:** 10.1186/s12947-015-0007-6

**Published:** 2015-03-21

**Authors:** Bernd Hewing, Sebastian Schattke, Sebastian Spethmann, Wasiem Sanad, Sabrina Schroeckh, Ingolf Schimke, Fabian Halleck, Harm Peters, Lars Brechtel, Jürgen Lock, Gert Baumann, Henryk Dreger, Adrian C Borges, Fabian Knebel

**Affiliations:** Department of Cardiology and Angiology, Charité-Universitätsmedizin Berlin, Campus Mitte, Charitéplatz 1, Berlin, 10117 Germany; Department of Nephrology, Charité-Universitätsmedizin Berlin, Campus Mitte, Berlin, Germany; Department of Medical Chemistry und Pathochemistry, Charité-Universitätsmedizin Berlin, Berlin, Germany; SMS Medical Institute, Berlin, Germany; Berlin Academy for Sport Medicine, Berlin, Germany; SCC Running Events GmbH, Berlin, Germany; Department of Medicine I, Cardiology, Helios Klinikum Emil von Behring, Berlin, Germany

**Keywords:** Athlete’s heart, Endurance exercise, Natriuretic peptides, Diastolic function, Renal function

## Abstract

**Background:**

Participation of amateur runners in endurance races continues to increase. Previous studies of marathon runners have raised concerns about exercise-induced myocardial and renal dysfunction and damage. In our pooled analysis, we aimed to characterize changes of cardiac and renal function after marathon running in a large cohort of mostly elderly amateur marathon runners.

**Methods:**

A total of 167 participants of the BERLIN-MARATHON (female n = 89, male n = 78; age = 50.3 ± 11.4 years) were included and cardiac and renal function was analyzed prior to, immediately after and 2 weeks following the race by echocardiography and blood tests (including cardiac troponin T, NT-proBNP and cystatin C).

**Results:**

Among the runners, 58% exhibited a significant increase in cardiac biomarkers after completion of the marathon. Overall, the changes in echocardiographic parameters for systolic or diastolic left and right ventricular function did not indicate relevant myocardial dysfunction. Notably, 30% of all participants showed >25% decrease in cystatin C-estimated glomerular filtration rate (GFR) from baseline directly after the marathon; in 8%, we observed a decline of more than 50%. All cardiac and renal parameters returned to baseline ranges within 2 weeks after the marathon.

**Conclusions:**

The increase in cardiac biomarkers after completing a marathon was not accompanied by relevant cardiac dysfunction as assessed by echocardiography. After the race, a high proportion of runners experienced a decrease in cystatin C-estimated GFR, which is suggestive of transient, exercise-related alteration of renal function. However, we did not observe persistent detrimental effects on renal function.

**Electronic supplementary material:**

The online version of this article (doi:10.1186/s12947-015-0007-6) contains supplementary material, which is available to authorized users.

## Background

Participation of non-elite, recreational runners (including elderly participants) in long-distance running events such as full or half marathons has increased in recent years. This trend might in parts reflect the increasing awareness among the general population that physical activity reduces cardiovascular risk and mortality [1]. However, although sport related deaths occur very rarely, several previous studies have reported detrimental, exercise-induced effects on myocardial function as assessed by echocardiography, cardiac magnet resonance imaging (CMR) or elevated cardiac biomarkers such as troponin or NT-proBNP after running a marathon [2-7]. It is not fully determined yet whether changes in cardiac parameters solely represent a transient physiological response to the exercise or may even signify persistent cardiac structural changes and dysfunction [8,9]. Similarly, studies have evaluated changes of renal function in athletes including long-distance runners. Transient increases of serum creatinine, cystatin C and urea nitrogen, indicating renal dysfunction, have been described after completion of a marathon [6,10,11].

In 2006 and 2007 we examined 78 male and 89 female, mostly elderly, amateur marathon runners by echocardiography and laboratory testing. First, we compared young versus old (≥60 years) male runners and were able to show that systolic function is preserved in both age groups after completion of a marathon and transient alterations of diastolic function over the race do not differ significantly between the two groups [8]. Secondly, we compared cardiac function of pre- versus postmenopausal female runners and found an improvement of left and right ventricular systolic function after completion of the marathon in both groups [9].

In the present analysis of all 167 amateur marathon runners we aimed to further characterize the overall changes of cardiac and renal function after marathon running in the pooled cohort of marathon runners to prove whether marathon running leads to acute or sustained cardiac or renal dysfunction. In addition, we evaluated the impact of training mileage on changes in cardiac function.

## Methods

All data were collected during our marathon studies in 2006 and 2007 as described previously [8,9].

### Study design

The organizers of the 2006 and 2007 BERLIN-MARATHON invited all registered male (2006) and female (2007) contestants in all age-groups from the Berlin-Brandenburg area by e-mail to participate in our study. The first 88 positive responses from male and 111 positive responses from female runners who had previously completed at least one marathon were screened and enrolled in the study. The maximum number of study participants in each year was limited by the logistics situation immediately after the races. Written informed consent was obtained from each participant. The Ethics Committee of the Charité-Universitätsmedizin Berlin hospital approved the study protocol. The study complied with the Declaration of Helsinki.

Exclusion criteria were recent pathological results from a previous bicycle stress test (bicycle stress test was mandatory for inclusion of participants older than 50 years), history or symptoms of coronary artery disease (e.g. angina pectoris or shortness of breath) or chronic cardiovascular disorders (atrial fibrillation, permanent pacemaker, bypass surgery, prosthetic valves or congenital heart disease).

The participants were examined at least 10 days prior to the marathon at rest by a questionnaire, blood test, blood pressure and heart rate measurements, ECG and echocardiography (baseline, pre). The questionnaire comprised detailed questions on running experience, previous completed marathons, average training kilometers per week, other sporting activities, present and past medical history (including chronic diseases, physical injuries, previous hospitalizations and surgeries), allergies, alcohol consumption, medications, cardiovascular risk factors such as history of smoking or family history of cardiovascular disease/risk factors. All subjects were advised to suspend training for at least 2 days before the baseline examination. Immediately after the marathon, runners were examined in a medical tent 100 m behind the finish line by a blood test and echocardiography (post); notably, the examinations of each individual runner were completed within approximately 20 minutes after the runner crossed the finish line with subsequent offline analysis of the digitally stored echocardiographic data (EchoPac PC, GE Vingmed, Horton, Norway). A follow-up examination was performed two weeks after the marathon at rest including blood test and echocardiography.

Thirty-two runners were excluded from the study for the following reasons: positive bicycle stress test results (4); a troponin T (TnT) level above the lower limit of detection (LLD) not explained by profound exercise training prior to sampling (2); uncontrolled arterial hypertension (1); hypermobile interatrial septum (1); premature ventricular contractions (2); history of a recent stroke (1); gynaecological operation before the marathon (1); anaemia due to resistance to common therapies treated with erythropoietin (1); leg cramps (1) or acute febrile disease, any of which led to non-attendance of the marathon race (5); and personal constraints in 6 runners. One runner did not reach the finish line, sufficient blood samples for all designated analyses could not be drawn from six athletes after the marathon (after 3 unsuccessful attempts by a medical assistant). Runners with positive baseline troponin or stress tests were encouraged to undergo further cardiac diagnostics. Finally, a total of 167 healthy male (n = 78) and female (n = 89) marathon runners were included.

There were no fluid or pace restrictions for the runners during the race.

### Biochemical studies

Blood samples were collected in a supine position from cubital veins at the time points mentioned above. EDTA blood was collected for haematological parameters and measured immediately after collection. For the other markers, serum was prepared by centrifugation, immediately frozen and stored at −20°C until processing. None of the specimens demonstrated signs of haemolysis. Laboratory results from the post-marathon time points were corrected intra-individually for dehydration, as previously described [12]. The change in plasma volume between the pre- and post-marathon time points was calculated using the method of Dill and Costill [12]. Cardiac troponin T (cTnT) measurements were performed using an Elecsys-2010 bench top analyser with the fourth generation assay (Roche Diagnostics GmbH, Mannheim, Germany). The LLD is 10 pg/mL, which is equal to the 99th percentile of the reference population [13]. NT-proBNP measurements were performed using an Elecsys-2010 bench top analyser (Elecsys proBNP, Roche Diagnostics, Mannheim, Germany). Age-adjusted cut-off values were set according to Hess et al. [14]. Serum cystatin C levels were determined using a particle-enhanced nephelometric immunoassay according to the manufacturer’s instructions (Dade Behring, Marburg, Germany). Glomerular filtration rate (GFR) was estimated using the following equation: estimated GFR (mL/min) = 74.835 / cystatin C (mg/L)^1.333^ [15].

### Echocardiography and Doppler measurements

Echocardiography was performed by experienced physicians of the echocardiography laboratory of the Cardiology Department, Charité-Universitätsmedizin Berlin, Germany. The echocardiographic parameters were obtained in the left decubitus position according to the guidelines of the American Society of Echocardiography (ASE) [16,17] using Vivid 7 Dimension (pre-race and follow-up echocardiograms) and portable Vivid-i ultrasound machines (post-race echocardiograms) (GE Vingmed, Horton, Norway, M3S 1.5-4.0 MHz transducer). Three beats were stored digitally and analyzed offline (EchoPac PC, GE Vingmed). Left heart dimensions were acquired by M-mode echocardiography or directly from 2D images according to Lang et al. [16]. LV mass was calculated using the Devereux formula and was indexed to the calculated body surface area using the Mosteller formula [16,18]. Left ventricular ejection fraction (LVEF) was estimated by Simpson’s biplane approach. Right heart dimensions were obtained according to Rudski et al. [17]. The frame rate for tissue Doppler (TDI) measurements was >100/s. For 2D strain analysis, frame rates of 60 to 80/s were used. Transmitral pw-Doppler inflow at the tips of the mitral leaflets was measured to obtain E, E deceleration time (DT), A and E/A ratio [19]. 2D strain variables and TDI measurements were assessed in the apical 4-chamber view. Peak early diastolic (E’), late diastolic (A’) and systolic (S’) velocities were measured at the basal septum [20,21]. The position of the sample volume for velocity and TDI strain measurements was manually positioned in the myocardium throughout the cardiac cycle. The LV and RV myocardial performance index (MPI) were determined as markers of global myocardial function of each chamber [22,23]. Tricuspid annular plane systolic excursion (TAPSE) was acquired by M-mode echocardiography according to Kaul et al. [24] and the longitudinal velocity of excursion (RV S’) was assessed by pulsed-wave TDI placed in the tricuspid annulus [17].

### Statistical analysis

Results are generally expressed as mean value ± standard deviation (SD) for normally distributed data or as median with interquartile range (IQR = 25th - 75th percentile) for non-normally distributed data. Assumption of normal distribution of data was verified by the Shapiro-Wilk test. The Mann–Whitney U-test was used for comparison of two independent groups and the Wilcoxon-test for comparison of paired observations. Correlations were calculated with the Spearman’s rank correlation coefficient. Frequencies of various groups were compared with chi-square test. Comparisons of changes in parameters at the pre- and post-marathon time points of the individual training groups were analyzed with pre-marathon variables as covariates. Statistical analyses were performed using SPSS 20.0 (SPSS Inc.) and SAS 9.2 (Statistical Analysis System Institute Inc.) software; p < 0.05 was considered statistically significant.

## Results

None of the participating 167 marathon runners had relevant medical problems during or immediately after the race. General baseline characteristics of the subjects are shown in Table 1. There was a significant increase of hemoglobin, hematocrit, protein and sodium in the blood immediately after the marathon indicating dehydration (Table 2). Accordingly, the median calculated reduction in plasma volume between the pre- and post-marathon time points was -6.4% (IQR -9.8% to -3.0%). Levels of CRP were within the normal range immediately after the race, but significantly lower compared to baseline values (Table 3).

**Table 1:** **General characteristics of all study participants**

Age [years]	50.3 (range: 22 - 72)
Gender [*n*], (%)	
Male	78 (47)
Female	89 (53)
Body mass index [kg/m^2^]	22.4 ± 2.1
Blood pressure [mmHg]	
Systolic	125.0 (120.0 - 130.0)
Diastolic	80.0 (75.0 - 85.0)
Pre-marathon heart rate [per min]	61.8 ± 9.0
Post-marathon heart rate [per min]	88.2 ± 14.2
Average training [km/week] (for at least 3 months before the marathon)	50.0 (40.0 - 65.0)
Long-distance running experience [years]	10.0 (6.0 - 20.0)
Previous marathons [*n*]	6.0 (3.0 - 13.0)
Running time [min]	263.0 ± 37.1

Values are shown as mean ± SD or median (IQR), except for age and gender.

**Table 2:** **Hemoglobin, hematocrit, protein and sodium of all study participants before (pre), immediately after (post) and 14 days after (follow-up) the marathon**

**Parameter**	**Pre**	**Post**	**Follow-up**	***p (Pre vs. Post)***	***p (Pre vs. Follow-up)***
**Hemoglobin** [mg/dl]	13.8 (13.1 - 14.3)	14.5 (13.8 - 15.6)	14.1 (13.4 - 15.0)	***<0.001***	***<0.001***
**Hematocrit**	0.40 (0.39 - 0.42)	0.42 (0.40 - 0.45)	0.42 (0.40 - 0.44)	***<0.001***	***<0.001***
**Protein** [g/dl]	7.2 (6.9 - 7.5)	8.0 (7.7 - 8.2)	7.4 (7.2 - 7.7)	***<0.001***	***<0.001***
**Sodium** [mmol/l]	140 (139 - 141)	143 (141 - 145)	140 (138 - 141)	***<0.001***	*0.893*

Values are shown as median (IQR). Statistically significant values are marked in bold.

**Table 3:** **C-reactive protein (CRP) and N-terminal prohormone of brain natriuretic peptide (NT-proBNP) of all study participants before (pre), immediately after (post) and 14 days after (follow-up) the marathon**

**Parameter**	**Pre**	**Post**	**Follow-up**	***p (Pre vs. Post)***	***p (Pre vs. Follow-up)***
**CRP** [mg/dl]	0.10 (0.05 - 0.21)	0.06 (0.04 - 0.12)	0.10 (0.06 - 0.18)	***<0.001***	*0.256*
**NT-proBNP** [pg/ml]	71.9 (41.2 - 124.1)	135.3 (77.7 - 219.8)	51.5 (30.5 - 82.2)	***<0.001***	***<0.001***

Values are shown as median (IQR). Statistically significant values are marked in bold.

### Echocardiography

At baseline, the average septal and posterior wall thicknesses, left atrial and ventricular diameter and LV mass index were within normal ranges (Table 4) and did not correlate significantly with the average training level or the numbers of previously completed marathons. All runners had a LVEF within the normal range (>55%).

**Table 4:** **Two-dimensional echocardiographic baseline data of all study participants**

IVSD [mm]	10.0 (9.0 - 11.0)
LVPWD [mm]	10.0 (9.0 - 11.0)
LVEDD [mm]	46.2 ± 4.9
LVESD [mm]	27.5 ± 4.9
LV mass index [g/m^2^]	
Male	109.8 (97.0 - 125.9)
Female	82.7 (74.3 - 96.7)
LA diameter [mm]	32.1 ± 4.0

Values are shown as mean ± SD or median (IQR). IVSD, septal wall thickness; LV, left ventricular; LVPWD, LV posterior wall thickness; LVEDD, LV end-diastolic diameter; LVESD, LV end-systolic diameter; LA, left atrial.

Immediately after the marathon, there was a significant increase in fractional shortening (FS), longitudinal 2D LV strain in the septal basal segment and LV S’ compared to baseline. The LV MPI remained unchanged compared to baseline (Table 5).

**Table 5:** **Echocardiographic variables of all study participants before (pre), immediately after (post) and 14 days after (follow-up) the marathon**

**Parameter**	**Pre**	**Post**	**Follow-up**	***p (Pre vs. Post)***	***p (Pre vs. Follow-up)***
***LV systolic function***					
FS [%]	41.5 (37.0-46.5)	46.0 (40.9-52.0)	45.0 (37.0-50.0)	***<0.001***	***0.001***
MPI LV	0.46 (0.40-0.55)	0.47 (0.41-0.58)	0.44 (0.35-0.52)	*0.116*	***0.018***
Longitudinal 2D strain septal basal [%]	-17.8 (15.5-20.2)	-19.6 (16.3-22.8)	-18.6 (15.6-21.4)	***<0.001***	*0.566*
Peak systolic velocity septal basal S’[m/s]	0.07 (0.07-0.08)	0.09 (0.08-0.10)	0.08 (0.07-0.09)	***<0.001***	***0.009***
***LV diastolic function***					
E [m/s]	0.80 (0.70-0.92)	0.67 (0.55-0.77)	0.81 (0.69-0.92)	***<0.001***	*0.281*
A [m/s]	0.60 (0.50-0.70)	0.69 (0.60-0.81)	0.60 (0.50-0.68)	***<0.001***	*0.531*
E/A	1.40 (1.10-1.60)	0.91 (0.76-1.20)	1.3 (1.10-1.67)	***<0.001***	*0.768*
E/E’	7.75 (6.63-9.43)	9.08 (7.41-11.08)	7.80 (6.87-9.15)	***0.001***	*0.150*
E’ septal [m/s]	0.10 (0.09-0.12)	0.09 (0.07-0.11)	0.10 (0.09-0.12)	***<0.001***	*0.484*
A’ [m/s]	0.09 (0.07-0.10)	0.11 (0.09-0.13)	0.09 (0.08-0.11)	***<0.001***	*0.070*
DT [ms]	187.0 (145.3-230.0)	130.0 (106.8-170.5)	177.0 (143.0-223.5)	***<0.001***	*0.324*
***Right heart parameters***					
RVEDD [mm]	32.0 (29.0-38.0)	31.0 (28.0-35.5)	34.0 (30.0-38.0)	***0.008***	*0.079*
TAPSE [mm]	28.0 (26.0-31.0)	28.0 (25.0-29.0)	28.0 (25.0-30.0)	***0.025***	*0.429*
RV S’ [m/s]	0.11 (0.10-0.12)	0.11 (0.09-0.13)	0.11 (0.10-0.13)	*0.958*	*0.325*
MPI RV	0.53 (0.41-0.72)	0.50 (0.41-0.65)	0.48 (0.38-0.71)	*0.196*	***0.004***
Longitudinal TDI RV strain basal [%]	-24.8 (20.0-29.5)	-23.6 (18.8-29.5)	-26.0 (21.1-30.1)	*0.681*	*0.197*
Longitudinal TDI RV strain mid [%]	-32.6 (26.7-39.1)	-29.0 (22.5-34.5)	-32.0 (24.1-37.9)	***<0.001***	*0.477*
Longitudinal TDI RV strain apical [%]	-32.4 (24.5-32.8)	-26.9 (20.5-32.8)	-27.8 (21.7-35.9)	***<0.001***	***0.020***

Values are shown as median (IQR). Statistically significant values are marked in bold. LV, left ventricular; FS, fractional shortening; MPI, myocardial performance index; E, peak transmitral E-wave velocity; A, peak transmitral A-wave velocity; E/A, ratio of transmitral E to transmitral A; E’ septal, early diastolic annular velocity measured in the septal annulus; A’, late diastolic annular velocity measured in the septal annulus; E/E’, ratio of peak early transmitral diastolic velocity to early septal annular velocity; DT, deceleration time of the transmitral E-wave; RV, right ventricular; RVEDD, RV end diastolic diameter; TAPSE, tricuspid annular plane systolic excursion; RV S’, peak systolic velocity of the basal RV free wall segment.

LV diastolic function parameters – E, DT and E’ – decreased significantly, while A and A’ increased. Accordingly, the E/A ratio decreased significantly after the race to an average value of 1.0 ± 0.4, while the E/E’ ratio as a marker of LV filling pressure increased significantly to an average of 9.0 ± 3.2. All diastolic function parameters returned to ranges of baseline levels within the follow-up period (Table 5).

The RV end diastolic diameter (RVEDD) was significantly reduced immediately after the marathon and the reduction in plasma volume was weakly correlated with the decrease of RVEDD (Spearman ρ = 0.178, p = 0.027). The right-heart function as assessed by TDI-derived RV S’, strain of the basal RV wall segment and the global function parameter MPI remained unchanged after the marathon. There was a decrease of TAPSE and strain of the mid and apical RV wall segments (Table 5), which reached statistical significance, but is most likely clinically irrelevant as values remained within the normal range. We were not able to systematically determine systolic PAP in our study population, since only a minority of the runners had detectable tricuspid regurgitation.

### Cardiac biomarkers

All runners (except two) had baseline cTnT levels below the lower limit of detection (LLD). The increased cTnT levels in the two runners were associated with intensive training immediately prior to blood sampling; there were no clinical, electrocardiographic or echocardiographic signs of an acute coronary syndrome. In 37.2% of all runners, cTnT increased to values above LLD/99th percentile immediately after the marathon, but returned to values below the LLD within the follow-up period. Runners with elevated post-marathon cTnT did not differ with respect to weekly training (no cTnT elevation: 52.6 ± 17.8 km, elevated cTnT: 54.9 ± 19.8 km, p = 0.476) or running time (no cTnT elevation: 264.6 ± 36.8 min, elevated cTnT: 260.4 ± 37.8 min, p = 0.406), CK levels (no cTnT elevation: 327.0 ± 185.3 U/l, elevated cTnT: 306.8 ± 247.5 U/l, p = 0.311) or any post-marathon echocardiographic variable – except for RVEDD, which was significantly lower in the elevated cTNT group (no cTnT elevation: 32.8 ± 5.3 mm, elevated cTnT: 30.6 ± 5.3 mm, p = 0.004). Furthermore, runners with elevated cTnT levels tended to be younger (no cTnT elevation: 51.8 ± 10.7 years, cTnT elevation: 47.6 ± 12.2 years, p = 0.045). There was no significant correlation of changes of cTNT and CRP levels between the pre- and post-marathon time points.

Overall, 34.1% of the runners demonstrated an increase of NT-proBNP above the age-adjusted cut-off value [14] after the marathon (Table 3). Runners with elevated post-marathon NT-proBNP levels did not differ with regards to average weekly training (no NT-proBNP elevation: 53.4 ± 18.1 km, elevated NT-proBNP: 53.2 ± 19.9 km, p = 0.842), running time (no NT-proBNP elevation: 261.8 ± 37.1 min, elevated NT-proBNP: 264.8 ± 35.3 min, p = 0.659), age (no NT-proBNP elevation: 50.8 ± 11.2 years, elevated NT-proBNP: 49.4 ± 12.2 years, p = 0.544) or any echocardiographic variable directly after the race, except for LV S’, which was lower (but within the normal range) in the elevated NT-proBNP group (no NT-proBNP elevation: 0.10 ± 0.02 m/s, elevated NT-proBNP: 0.09 ± 0.02 m/s, p = 0.002). There was no significant correlation of changes of NT-proBNP and CRP levels between the pre- and post-marathon time points.

13.4% of the runners had both NT-proBNP and cTnT elevations. However, there was no significant correlation between elevated NT-proBNP and cTNT levels after the marathon (chi-square test, p = 0.735).

### Renal function

Serum creatinine and cystatin C levels increased significantly immediately after the marathon (Table 6). Accordingly, the calculated cystatin C-based GFR was significantly decreased after the marathon. According to the RIFLE classification for acute kidney injury (AKI) [25], 30.3% of the runners showed a decrease of GFR between >25% and ≤50% representing the risk stratum, and 8.4% of the runners demonstrated a decrease of GFR by >50% signifying the injury stratum. All parameters returned to baseline ranges within the 2 weeks follow-up. There was a weak but significant correlation of the changes of cystatin C between the pre- and post-marathon time points with running time (Spearman ρ = -0.307, p <0.001), changes of NT-proBNP (Spearman ρ = 0.279, p <0.001) and cTNT (Spearman ρ = 0.142, p <0.002), but not with age, average weekly training or changes of CRP and CK.

**Table 6:** **Creatinine, cystatin C and cystatin C-estimated GFR before (pre), immediately after (post) and 14 days after (follow-up) the marathon (** ***n*** **= minimum of 155 study participants)**

**Parameter**	**Pre**	**Post**	**Follow-up**	**p (Pre vs. Post)**	**p (Pre vs. Follow-up)**
**Creatinine** [mg/dL]	0.83 (0.75-0.93)	1.02 (0.88-1.26)	0.82 (0.72-0.92)	**<0.001**	**0.003**
**Cystatin C** [mg/dL]	0.68 (0.62-0.78)	0.85 (0.69-0.99)	0.66 (0.59-0.78)	**<0.001**	**<0.001**
**Cystatin C-estimated GFR** [mL/min]	125.1 (104.2-141.5)	93.7 (75.7-122.3)	130.2 (104.2-151.2)	**<0.001**	**<0.001**

Values are shown as median (IQR). Statistically significant values are marked in bold. GFR, glomerular filtration rate.

### Subgroup analysis

Based on their average weekly training in kilometers, all runners were divided into three groups according to Neilan et al. [26]: I) <56 km, II) 57 - 72 km and III) >72 km per week (see Additional file 1). Overall, changes in parameters of LV systolic and diastolic and right heart function did not differ significantly between groups except for E/E’, which reached statistical significance. All three groups did not differ significantly in the proportion of runners with elevated levels of cTnT (chi-square test, p = 0.630) or NT-proBNP (chi-square test, p = 0.164).

## Discussion

Increases of the cardiac biomarkers NT-proBNP and troponin can regularly be found after endurance exercise [27]. In our large cohort of marathon runners, over 50% of the runners exhibited an increase of at least one of the two biomarkers. The elevation of both biomarkers did not correlate with each other and was only temporary returning to normal ranges within the 2 weeks follow-up. In contrast to previous studies [26,28], we could not observe an association of cardiac biomarker elevation with training levels. Portions of marathon runners with positive cardiac troponin levels after the race differ between studies based on different troponin generations tested, time points of blood sampling, cut-off values used and whether samples were corrected for dehydration as in the present study. We found that 37% of the runners had an increase of fourth generation cTNT at or about the limit of detection for myocardial infarction. For the subgroup of male runners we have previously shown that, after applying a highly sensitive assay for cardiac troponin (hs cTNT), the percentage of runners with troponin over the 99th percentile cut-off (13 ng/L) increased to 94% [29]. The exact mechanisms of the biomarker release are not completely understood yet. Increased levels of circulating cTNT after exercise are considered to be unrelated to irreversible cardiac injury [30] and may result from an increased permeability of the cardiomyocyte membrane with subsequent leakage of cytosolic troponin due to altered metabolic conditions, transient inflammation and reversible ischemia [7,29]. Furthermore, we observed a weak but significant correlation between the changes in cystatin C levels and the increase of cTNT, thus indicating a potential role of renal elimination in exercise-induced increases of cTNT. NT-proBNP levels were increased above age-adjusted cut-off values in 34% of the runners, which however did not correlate with the average weekly training mileage as previously suggested [26]. Long-term clinical relevance of repetitive exercise-induced cTNT and NT-proBNP releases remains to be determined, but bearing in mind that endurance athletes have a longer life expectancy than the general population [31] substantial and prolonged exercise-induced myocardial damage may seem rather unlikely. Thus, more prospective studies with long-term follow-ups are urgently needed.

Baseline echocardiography of the study participants did not reveal typical characteristics of an athlete’s heart [32]. After the marathon, we observed a physiological increase in systolic LV function (FS, longitudinal 2D strain and S’) as a result of the increased inotropy. There was a transient alteration of LV diastolic function parameters (decreased E/A ratio, DT, E and E’ and increased A and A’) after the race most likely related to the apparent dehydration [33] and the increased heart rate [34,35]. The E/E’ ratio increased significantly (to an average of 9.0) immediately after the marathon (an E/E’ ratio of ≥15 indicates pathological filling pressures in humans with cardiac disease) [35]. However, the use of the E/E’ ratio for estimating filling pressures in healthy hearts is controversially discussed as the E/E’ ratio is preload dependent and may be disproportionally elevated in the presence of hypovolemia despite low filling pressures [36,35]. The average baseline E/E’ value of 8.0 in our study population reflects the age- and sex-dependent physiological adaptation of diastolic parameters in healthy individuals [37].

Previous studies on (ultra-) endurance athletes have suggested RV dysfunction and increases of RV volume after exercise [2,5,26]. However, in our study cohort we found no evidence of clinically relevant right heart myocardial dysfunction. Parameters of systolic and global RV function (RV S’, RV basal strain, MPI) remained unchanged, while TAPSE and strain of the mid and apical RV wall segments changed within normal, clinically non-relevant ranges. Furthermore, RVEDD decreased significantly in our study cohort after the race, which we primarily attribute to the apparent dehydration. In contrast to most of the above mentioned studies on endurance athletes, all post-marathon assessments were completed before rehydration was initiated. As a limitation it has to be critically considered that the diagnostic accuracy describing RV volume by 2D echocardiography is still not sufficiently validated.

We measured cystatin C for evaluation of renal filtration, which is synthesized constantly by all nucleated cells and is virtually independent of age, muscle mass and muscle work [11]. We found a significant increase in cystatin C levels after completion of the marathon in our study cohort which normalized within the 2 weeks follow-up. This transient alteration of renal function may be of pre-renal origin due to the hydration status of the runners after the race, but the impact of other aggravating factors such as exercise-induced inflammation or oxidative stress should be addressed in further studies. However, we did not observe an association of changes of cystatin C levels and CRP, NT-proBNP or CK after the race among our runners that would indicate a substantial inflammatory trigger, acute cardiorenal syndrome or rhabdomyolysis, respectively, as causes for the altered renal function. Furthermore, it remains to be proven whether repetitive, exercise-induced alterations of renal function lead to detrimental long-term effects in healthy runners and in runners with pre-existing renal impairment. However, we did not observe an association between average training levels and baseline creatinine or cystatin C in our study cohort that would support this hypothesis.

Our analysis has several limitations: although an identical study design was used for studies of male or female runners, they needed to be evaluated in different marathons (for logistical reasons), which inherits the bias of different climate conditions. Post-marathon blood pressure and weight were not recorded, which might have provided more detailed information on changes in hemodynamics, fluid balance and dehydration status. With regard to renal function and grading of AKI, we did not monitor urine output and composition over the race.

The present study comprises one of the largest cohorts of amateur marathon runners in whom both cardiac and renal function were evaluated by echocardiography and biomarkers. We did not detect relevant LV or RV systolic dysfunction after completion of the marathon. There was a transient alteration of LV diastolic function parameters after the marathon which was not associated with increases in cardiac biomarkers. NT-proBNP and/or cTnT transiently increased after the marathon in over half of the runners. Regarding renal function, there was a transient decrease in cystatin C-estimated GFR >25% in almost 40% of the runners. However, all cardiac and renal parameters returned to baseline ranges within the two weeks follow-up. Therefore, we conclude that running a marathon is not associated with persistent impairment of myocardial or renal function in amateur marathon runners. The impact of our findings on long-term outcomes should be addressed in future studies.

## Additional file

Additional file 1:
**Subgroup analysis of baseline (pre) and post-marathon (post) variables depending on average training before the marathon.**

## References

[1] Perk J, De Backer G, Gohlke H, Graham I, Reiner Z, Verschuren M (2012). European guidelines on cardiovascular disease prevention in clinical practice (version 2012). the fifth joint task force of the European society of cardiology and other societies on cardiovascular disease prevention in clinical practice (constituted by representatives of nine societies and by invited experts). Eur Heart J.

[2] La Gerche A, Burns AT, Mooney DJ, Inder WJ, Taylor AJ, Bogaert J (2012). Exercise-induced right ventricular dysfunction and structural remodelling in endurance athletes. Eur Heart J.

[3] O'Keefe JH, Patil HR, Lavie CJ, Magalski A, Vogel RA, McCullough PA (2012). Potential adverse cardiovascular effects from excessive endurance exercise. Mayo Clin Proc.

[4] Scharhag J, George K, Shave R, Urhausen A, Kindermann W (2008). Exercise-associated increases in cardiac biomarkers. Med Sci Sports Exerc.

[5] Oxborough D, Shave R, Warburton D, Williams K, Oxborough A, Charlesworth S (2011). Dilatation and dysfunction of the right ventricle immediately after ultraendurance exercise: exploratory insights from conventional two-dimensional and speckle tracking echocardiography. Circ Cardiovasc Imaging.

[6] Scherr J, Braun S, Schuster T, Hartmann C, Moehlenkamp S, Wolfarth B (2011). 72-h kinetics of high-sensitive troponin T and inflammatory markers after marathon. Med Sci Sports Exerc.

[7] Shave R, Baggish A, George K, Wood M, Scharhag J, Whyte G (2010). Exercise-induced cardiac troponin elevation: evidence, mechanisms, and implications. J Am Coll Cardiol.

[8] Knebel F, Schimke I, Schroeckh S, Peters H, Eddicks S, Schattke S (2009). Myocardial function in older male amateur marathon runners: assessment by tissue Doppler echocardiography, speckle tracking, and cardiac biomarkers. J Am Soc Echocardiogr.

[9] Knebel F, Spethmann S, Schattke S, Dreger H, Schroeckh S, Schimke I (2012). Exercise-induced changes of left ventricular diastolic function in postmenopausal amateur marathon runners: assessment by echocardiography and cardiac biomarkers. Eur J Prev Cardiol.

[10] McCullough PA, Chinnaiyan KM, Gallagher MJ, Colar JM, Geddes T, Gold JM (2011). Changes in renal markers and acute kidney injury after marathon running. Nephrol (Carlton).

[11] Mingels A, Jacobs L, Kleijnen V, Wodzig W, Dieijen-Visser M (2009). Cystatin C a marker for renal function after exercise. Int J Sports Med.

[12] Dill DB, Costill DL (1974). Calculation of percentage changes in volumes of blood, plasma, and red cells in dehydration. J Appl Physiol.

[13] Apple FS, Quist HE, Doyle PJ, Otto AP, Murakami MM (2003). Plasma 99th percentile reference limits for cardiac troponin and creatine kinase MB mass for use with European society of cardiology/American college of cardiology consensus recommendations. Clin Chem.

[14] Hess G, Runkel S, Zdunek D, Hitzler WE (2005). Reference interval determination for N-terminal-B-type natriuretic peptide (NT-proBNP): a study in blood donors. Clin Chim Acta.

[15] Thomas L, Huber AR (2006). Renal function–estimation of glomerular filtration rate. Clin Chem Lab Med.

[16] Lang RM, Bierig M, Devereux RB, Flachskampf FA, Foster E, Pellikka PA (2005). Recommendations for chamber quantification: a report from the American society of Echocardiography’s guidelines and standards committee and the chamber quantification writing group, developed in conjunction with the European association of echocardiography, a branch of the European society of cardiology. J Am Soc Echocardiogr.

[17] Rudski LG, Lai WW, Afilalo J, Hua L, Handschumacher MD, Chandrasekaran K (2010). Guidelines for the echocardiographic assessment of the right heart in adults: a report from the American society of echocardiography endorsed by the European association of echocardiography, a registered branch of the European society of cardiology, and the Canadian society of echocardiography. J Am Soc Echocardiogr.

[18] Mosteller RD (1987). Simplified calculation of body-surface area. N Engl J Med.

[19] Mantero A, Gentile F, Azzollini M, Barbier P, Beretta L, Casazza F (1998). Effect of sample volume location on Doppler-derived transmitral inflow velocity values in 288 normal subjects 20 to 80 years old: an echocardiographic, two-dimensional color Doppler cooperative study. J Am Soc Echocardiogr.

[20] Sohn DW, Chai IH, Lee DJ, Kim HC, Kim HS, Oh BH (1997). Assessment of mitral annulus velocity by Doppler tissue imaging in the evaluation of left ventricular diastolic function. J Am Coll Cardiol.

[21] Kim YJ, Sohn DW (2000). Mitral annulus velocity in the estimation of left ventricular filling pressure: prospective study in 200 patients. J Am Soc Echocardiogr.

[22] Bruch C, Schmermund A, Marin D, Katz M, Bartel T, Schaar J (2000). Tei-index in patients with mild-to-moderate congestive heart failure. Eur Heart J.

[23] Tei C, Dujardin KS, Hodge DO, Bailey KR, McGoon MD, Tajik AJ (1996). Doppler echocardiographic index for assessment of global right ventricular function. J Am Soc Echocardiogr.

[24] Kaul S, Tei C, Hopkins JM, Shah PM (1984). Assessment of right ventricular function using two-dimensional echocardiography. Am Heart J.

[25] Bellomo R, Ronco C, Kellum JA, Mehta RL, Palevsky P (2004). Acute renal failure - definition, outcome measures, animal models, fluid therapy and information technology needs: the second international consensus conference of the acute dialysis quality initiative (ADQI) group. Crit Care.

[26] Neilan TG, Januzzi JL, Lee-Lewandrowski E, Ton-Nu TT, Yoerger DM, Jassal DS (2006). Myocardial injury and ventricular dysfunction related to training levels among nonelite participants in the Boston marathon. Circulation.

[27] Shave R, George KP, Atkinson G, Hart E, Middleton N, Whyte G (2007). Exercise-induced cardiac troponin T release: a meta-analysis. Med Sci Sports Exerc.

[28] Neilan TG, Yoerger DM, Douglas PS, Marshall JE, Halpern EF, Lawlor D (2006). Persistent and reversible cardiac dysfunction among amateur marathon runners. Eur Heart J.

[29] Saravia SG, Knebel F, Schroeckh S, Ziebig R, Lun A, Weimann A (2010). Cardiac troponin T release and inflammation demonstrated in marathon runners. Clin Lab.

[30] Hickman PE, Potter JM, Aroney C, Koerbin G, Southcott E, Wu AH (2010). Cardiac troponin may be released by ischemia alone, without necrosis. Clin Chim Acta.

[31] Teramoto M, Bungum TJ (2010). Mortality and longevity of elite athletes. J Sci Med Sport.

[32] Prior DL, La Gerche A (2012). The athlete’s heart. Heart.

[33] Abali G, Tokgozoglu L, Ozcebe OI, Aytemir K, Nazli N (2005). Which Doppler parameters are load independent? A study in normal volunteers after blood donation. J Am Soc Echocardiogr.

[34] Burns AT, Connelly KA, La Gerche A, Mooney DJ, Chan J, MacIsaac AI (2007). Effect of heart rate on tissue Doppler measures of diastolic function. Echocardiography.

[35] Nagueh SF, Appleton CP, Gillebert TC, Marino PN, Oh JK, Smiseth OA (2009). Recommendations for the evaluation of left ventricular diastolic function by echocardiography. Eur J Echocardiogr.

[36] Jacques DC, Pinsky MR, Severyn D, Gorcsan J (2004). Influence of alterations in loading on mitral annular velocity by tissue Doppler echocardiography and its associated ability to predict filling pressures. Chest.

[37] Mogelvang R, Sogaard P, Pedersen SA, Olsen NT, Schnohr P, Jensen JS (2009). Tissue Doppler echocardiography in persons with hypertension, diabetes, or ischaemic heart disease: the Copenhagen City Heart Study. Eur Heart J.

