# Supplementary material for: Cardiac and renal function in a large cohort of amateur marathon runners
Source: Cardiovasc Ultrasound. 2015 Mar 21;13:13. doi: 10.1186/s12947-015-0007-6 (PMC4372316; doi:10.1186/s12947-015-0007-6)
Supplement: Additional file 1: — Subgroup analysis of baseline (pre) and post-marathon (post) variables depending on average training before the marathon. [file 12947_2015_7_MOESM1_ESM.pdf]

Additional file 1. Subgroup analysis of baseline (pre) and post-marathon (post) variables depending on average training before the marathon

**Parameter**	**Group I ≤ 56 km/wk (n = 102)**	**Group II 57-72 km/wk (n = 40)**	**Group III > 72 km/wk (n = 25)**	**p inter-group**	**p intragroup Pre vs. Post**		
					**I**	**II**	**III**
**Age** [years]	50.3 ± 10.6	48.6 ± 10.7	52.9 ± 15.3	*0.70*			
**Male** (%)	40 %	42 %	80 %				
**Average training** [km/week]	42.5 (35.0-50.0)	65.0 (60.0-70.0)	85.0 (80.0-90.0)	***<0.001***			
**Finishing time** [min]	273.5 (249.5-302.0)	245.0 (216.0-270.0)	237.0 (214.0-260.0)	***<0.001***			
**Heart rate** [bpm]							
Pre	62.6 ± 8.7	60.5 ± 9.6	60.4 ± 9.4				
Post	88.0 ± 12.3	89.2 ± 16.5	87.7 ± 17.9	*0.08*	***<0.001***	***<0.001***	***<0.001***
**NT-proBNP** [pg/ml]							
Pre	70.2 (43.1-131.4)	73.5 (33.9-102.7)	78.6 (38.3-135.9)				
Post	146.7 (98.6-224.9)	107.3 (64.8-208.1)	128.0 (84.0-325.8)	*0.31*	***<0.001***	***<0.001***	***<0.001***
LV SYSTOLIC FUNCTION							
**MPI**							
Pre	0.47 (0.40-0.54)	0.46 (0.37-0.59)	0.46 (0.37-0.55)				
Post	0.47 (0.41-0.55)	0.50 (0.43-0.62)	0.46 (0.39-0.55)	*0.36*	*0.23*	*0.44*	*0.63*
**Longitudinal 2D strain septal basal** (%)							
Pre	-18.3 (15.5-20.4)	-17.3 (15.4-20.3)	-17.8 (15.8-19.6)				
Post	-20.2 (16.4-22.7)	-18.9 (16.2-22.9)	-17.4 (15.0-22.9)	*0.57*	***<0.001***	***<0.001***	*0.25*
**Peak systolic velocity septal basal S'**[m/s]							
Pre	0.07 (0.07-0.08)	0.08 (0.07-0.08)	0.07 (0.07-0.09)				
Post	0.09 (0.08-0.10)	0.10 (0.09-0.10)	0.09 (0.08-0.11)	*0.21*	***<0.001***	***<0.001***	***0.001***

LV DIASTOLIC FUNCTION							
**E** [m/s]							
Pre	0.79	0.84	0.75				
	(0.67-0.91)	(0.72-0.94)	(0.70-0.92)				
Post	0.66	0.72	0.61	*0.38*	***<0.001***	***0.01***	***0.011***
	(0.56-0.77)	(0.58-0.88)	(0.54-0.75)				
**A** [m/s]							
Pre	0.61	0.54	0.61				
	(0.50-0.60)	(0.48-0.61)	(0.51-0.71)				
Post	0.69	0.70	0.72	*0.84*	***<0.001***	***<0.001***	*0.13*
	(0.61-0.83)	(0.60-0.77)	(0.51-0.85)				
**E/A**							
Pre	1.40	1.44	1.20				
	(1.01-1.57)	(1.21-1.89)	(1.00-1.63)				
Post	0.90	1.10	0.90	*0.71*	***<0.001***	***<0.001***	***0.022***
	(0.74-1.16)	(0.80-1.24)	(0.70-1.14)				
**E/E'**							
Pre	7.73	7.83	7.56				
	(6.62-9.44)	(6.67-8.97)	(6.60-10.67)				
Post	9.04	9.62	9.00	***0.02****	***<0.001***	***<0.001***	0.672
	(7.21-10.97)	(7.83-13.11)	(5.89-10.86)				
**E' septal** [m/s]							
Pre	0.10	0.11	0.09				
	(0.09-0.12)	(0.09-0.13)	(0.07-0.11)				
Post	0.09	0.09	0.08	*0.78*	***0.001***	***0.003***	*0.34*
	(0.08-0.11)	(0.07-0.10)	(0.07-0.11)				
**A'** [m/s]							
Pre	0.09	0.08	0.08				
	(0.08-0.10)	(0.07-0.10)	(0.07-0.10)				
Post	0.11	0.11	0.11	*0.41*	***<0.001***	***<0.001***	*0.16*
	(0.09-0.13)	(0.09-0.13)	(0.09-0.14)				
**DT** [ms]							
Pre	201.4	171.5	155.5				
	(149.8-234.8)	(143.9-228.0)	(130.5-193.0)				
Post	126.0	132.0	132.0	*0.90*	***<0.001***	***0.001***	***0.006***
	(107.0-172.5)	(104.5-167.1)	(97.0-155.0)				

RIGHT HEART PARAMETERS							
**RVEDD** [mm]							
Pre	32.0<br>(28.3-37.0)	33.0<br>(30.0-38.0)	36.0<br>(30.3-39.8)				
Post	31.0<br>(28.0-35.0)	32.0<br>(27.0-35.0)	33.5<br>(28.3-39.0)	*0.52*	***0.025***	*0.21*	*0.47*
**TAPSE** [mm]							
Pre	28.0<br>(26.0-31.0)	28.0<br>(26.0-30.0)	29.5<br>(25.5-31.8)				
Post	28.0<br>(25.0-29.0)	28.0<br>(27.0-30.0)	29.0<br>(25.0-30.0)	*0.21*	***0.003***	*0.63*	*0.67*
**RV S'** [m/s]							
Pre	0.11<br>(0.10-0.12)	0.12<br>(0.10-0.14)	0.11<br>(0.09-0.12)				
Post	0.11<br>(0.10-0.13)	0.11<br>(0.08-0.13)	0.10<br>(0.09-0.13)	*0.26*	*0.18*	*0.08*	*0.84*

** p = 0.53 for group I vs. II, p = 0.02 for group I vs. III, p = 0.02 for group II vs. III*

Values are shown as mean ± SD or median (IQR). NT-proBNP, N-terminal prohormone of brain natriuretic peptide; LV, left ventricular; MPI, Myocardial performance index; E, peak transmitral E-wave velocity; A, peak transmitral A-wave velocity; E/A, ratio of transmitral E to transmitral A; E' septal, early diastolic annular velocity measured in the septal annulus; A', late diastolic annular velocity measured in the septal annulus; E/E', ratio of peak early transmitral diastolic velocity to early septal annular velocity; DT, deceleration time of the transmitral E-wave; RV, right ventricular; RVEDD, RV end diastolic diameter; TAPSE, tricuspid annular plane systolic excursion; RV S', peak systolic velocity of the basal RV free wall segment.
